# Exposure of Children to Unhealthy Food and Beverage Advertisements in South Africa

**DOI:** 10.3390/ijerph18083856

**Published:** 2021-04-07

**Authors:** Daniel A. Yamoah, Jeroen De Man, Sunday O. Onagbiye, Zandile J. Mchiza

**Affiliations:** 1 School of Public Health, University of the Western Cape, Bellville 7535, South Africa; 3755458@myuwc.ac.za; 2 Department of Family Medicine and Population Health, University of Antwerp, 2610 Antwerp, Belgium; Jeroen.DeMan@uantwerpen.be; 3 Department of Sport, Recreation and Exercise Science, University of the Western Cape, Bellville 7535, South Africa; sonagbiye@uwc.ac.za

**Keywords:** unhealthy food advertisement, obesity, children, South Africa, self-regulation, persuasive techniques, alcohol, food marketers

## Abstract

Television (TV) is a powerful medium for marketing food and beverages. Food and beverage marketers tend to use this medium to target children with the hope that children will in turn influence their families’ food choices. No study has assessed the compliance of TV marketers with the South African Marketing to Children pledge since the enactment of the 2014 food advertising recommendations by the South African Department of Health and the Advertising Standards Authority. This study investigated the extent and nature of advertising of unhealthy versus healthy food and beverages to children in South African TV broadcasting channels. The date, time, type, frequency and target audience of food advertisements (ads) on four free-to-air South African TV channels were recorded and captured using a structured assessment guide. The presence of persuasive marketing techniques was also assessed. Unhealthy food and beverage advertising was recorded at a significantly higher rate compared with healthy food and beverages during the time frame when children were likely to be watching TV. Brand benefit claims, health claims and power strategies (e.g., advertising using cartoon characters and celebrated individuals) were used as persuasive strategies. These persuasive strategies were used more in unhealthy versus healthy food ads. The findings are in breach of the South African Marketing to Children pledge and suggest a failure of the industry self-regulation system. We recommend the introduction of monitored and enforced statutory regulations to ensure healthy TV food advertising space.

## 1. Introduction

Childhood overweightness and obesity prevalence have increased at an alarming rate and have become most serious global health challenges [1]. South Africa is not immune to these childhood health challenges in that, according to the outcomes of the South African National Health and Nutrition Examination Survey (SANHANES-1), the prevalence of overweightness and obesity in children aged 10 to 14 years are 12.1 and 4.2 percent, respectively, while in those children aged 15 to 17 years are 13.3 and 4.8 percent, respectively [2]. Unhealthy food and beverage advertising on television (TV) has been implicated in the development of childhood overweightness and obesity [3]. For instance, TV food and beverage advertising exposure has been shown to influence the amount of food that children who watch a lot of TV consume [4,5]. The most advertised food and beverages on TV tend to be high in fat, sugar and salt and low in essential minerals, vitamins, amino acids and fibre [6]. Children seem to also be more susceptible than adults to the persuasive approach used by TV marketers when advertising food and beverages [4,7]. Hence, children who watch food and beverage advertisements (ads) tend to choose these foods and beverages thinking they are healthy with less interest in knowing their nutrient content [7,8].

Food marketers have typically used a mixture of techniques to increase children’s desire for unhealthy food and beverages [9,10]. An example of these techniques is the use of misleading claims that portray specific food and beverages as bringing about enhanced performance (e.g., in sport, at schools) [9]. Others have utilised cartoon-related characters that are known to increase brand recognition among children [11]. Advertisements also portray people who make unhealthy food choices to appear to have desirable outcomes [12].

Given the extensive evidence of the negative impact of food advertising on children, the World Health Organisation (WHO) advocated a control of TV food marketing especially with regard to marketing directed towards children [13,14]. This could support the creation of a food environment that promotes healthy dietary choices. The WHO also proposed that countries should develop and adopt policies to control the marketing of food towards children with a specific emphasis on the reach, frequency, creative content, design and execution of the marketing message [14]. The WHO has argued that policies initiated against the negative influence of marketing such as TV food advertising need to be comprehensive to be efficient [14].

In South Africa, the number of peer-reviewed studies investigating children’s exposure to unhealthy food ads is limited. A study conducted by Van Vuuran between 2003 and 2005 [15] estimated that children were exposed to a daily average of 24 minutes of advertising. Subsequently, the South African government proposed a better control of TV food advertising to children in response to the call by the WHO [16]. This led to the development of a code for advertising by the Department of Health and the major food corporation’s consortium [17,18]. The food and beverage advertising code was formally initiated by the Advertising Standards Authority [ASA] in 2008. This code led to a pledge (i.e., the South African Marketing to Children pledge) to adhere to the code signed by the members of the major food corporations in 2009. The core principle was to publicly pledge “to commit to marketing communications to children who are twelve years old and under, to promote healthy dietary choices and healthy lifestyles” [17]. This pledge, as the sole form of regulation to control food ads, put South Africa in the group where food industries regulate themselves (i.e., self-regulation). This type of regulation is widely known for non-compliance by food marketers [10,19].

It is therefore not surprising that even after signing the pledge, studies found a poor adherence to the TV advertising guidelines in South Africa with major infringements of the pledge being identified [20,21,22]. According to the aforementioned studies, unhealthy food advertising continues to be prevalent in South Africa. The most frequent ads shown during periods when children are likely to be watching TV are for desserts and sweets, fast foods, hot sugar-sweetened beverages, starchy foods and sweetened beverages as found by Mchiza et al. [21]. Additionally, 67% of alcohol-related ads are shown during family viewing time [21].

Based on these findings by Mchiza et al. [21], the following recommendations were made to the Department of Health (DoH) and the ASA of South Africa in 2014 [23]:

1. The prohibition of the advertising of foods and beverages high in fat, sugar and salt following the World Health Organization (WHO) recommendations;
2. The prohibition of alcohol ads especially when children are watching;
3. The restriction of the use of advertising techniques that appeal to children. Ads should not use cartoon characters and/or animations or include promotional offers and gifts or tokens.

No research has assessed the rate of advertising of unhealthy food and beverages on children since the enactment of the 2014 food advertising recommendations by the South African DoH and ASA. Furthermore, no study has investigated the compliance of food marketers with the South African Marketing to Children pledge [17]. To address these gaps, this study investigated the extent and nature of advertising of unhealthy versus healthy food and beverages to children by the major South African TV broadcasting channels.

## 2. Methods and Procedures

The categories and techniques employed in this study were adapted from the International Network for Food and Obesity/Non-Communicable Diseases Research, Monitoring and Action Support (INFORMAS) module relating to the monitoring and benchmarking of unhealthy food promotion to children [24]. This approach was evaluated as adequate to assess the frequency and level of exposure of population groups (especially children) to food promotions, the persuasive power of techniques used in promotional communications and the nutritional composition of promoted food products. The South African Nutrient Profiling Model (SA-NPM) was used for the contextual adaptation of the tool [25].

### 2.1. Channels and Time of Broadcasting

Food ads were recorded from the four major South African TV channels. Recordings were done from 15:00 to 19:00 (i.e., 4 h) for seven consecutive days from 23 April 2017 to 29 April 2017. This resulted in a total of 112 broadcasting hours for the four stations together.

### 2.2. Description of the Target Audience of Broadcasting Channels

Television in South Africa is funded from license fees and advertising and broadcasts on four free-to-air channels (South African Broadcasting Corporation (SABC) 1, 2, 3 and Enhanced Television (e-TV)) with a mixed entertainment and public service mandate. According to the SABC segmentation, all four TV channels focus on the same target audience during the following intervals. From 15:00–17:00 hours they target children; during this time, the child-focused programs shown are infomercials, educational programs and cartoons. Following this time, from 17:00–19:00 hours, the target becomes the whole family including children. During this period, talk shows and soap operas are shown (Table 1). For the sake of the current study, these two time periods form the stipulated period when children are expected to be part of the TV audience.

### 2.3. Selection and Coding Procedures

The data were collected manually by recording the live video broadcasted on the four TV stations concurrently within the stipulated period. A TV tuner (WinTV tuner), a Windows Media Centre compatible with Windows 10 and storage devices were the tools employed to carry out the task. Coding was done by two independent researchers (a nutrition expert with a PhD in nutrition and dietetics and a postgraduate researcher with a Master of Public Health specializing in nutrition). Both researchers independently viewed a playback of the recorded videos one TV station at a time. In the case of any disagreement, recoding was done until 100% agreement was reached. No distinction was made between unique and repetitive ads.

Ads were selected if they fell into one of the following categories and were coded accordingly: (i) healthy food or beverage, (ii) unhealthy food or beverage, (iii) neutral (Table 2). Healthy foods were defined as core foods that are nutrient-dense and recommended for daily consumption. Unhealthy foods were non-core foods that are high in undesirable nutrients such as fat, refined sugars and salt. The neutral category consisted of food and beverage-related items that could not explicitly be labelled as healthy or unhealthy such as baby and toddler milk formula, tea and coffee.

For each ad, the following information was collected: (i) television channel (TV station being recorded); (ii) name and type of program in which the ad was shown; (iii) date and time of the day when the ad was shown; (iv) assumed target audience of the ad; (v) company placing the ad; (vi) description of the product advertised; (vii) brand benefit claim (claims other than those relating to health that were directed towards developing positive perceptions about a company’s product that might influence brand attachment), if any; (viii) description of health claim (any claim of the food or its constituent having an effect on health or being healthy or having a nutritional property) [24], if any; (ix) power strategy (this could be a promotional character or event or person employed to increase the persuasive power of an advertisement) [24], if any; (x) duration of the ad. Brand benefit claims, health claims and power strategies constituted the persuasive techniques that were studied. This study focused only on child and family viewing times.

### 2.4. Statistical Analysis

A descriptive analysis was done using measures of central tendency, standard deviations (SD) and ad rates for different ad subgroups (e.g., TV channel, viewing time). The differences between the healthy and unhealthy categories were calculated using a 1-sample proportions test with a Yates’s continuity correction. For small samples, an exact binomial test was used. The analysis was done using R software [26].

### 2.5. Ethical Considerations

The Humanities and Social Science Research Ethics Committee of the University of the Western Cape approved the methodology and exempted the ethics of the current research (Reference Number: HS19/6/6). The project is also registered with the University of the Western Cape’s Higher Degrees Committee. The data did not include any personal information.

## 3. Results

### 3.1. General Description

A total of 1629 ads were shown on the four TV channels combined of which 582 (35.7%) related to food and beverages. This corresponded to an average advertisement rate of 5.2 ads/c-h. Unhealthy food/beverage items constituted more than half (342: 58.8%) of the total food/beverage-related ads followed by neutral ads (150: 25.8%) and healthy food/beverage ads (90: 15.5%). The mean duration of the ads was 29.4 s and ranged from 6 to 45 s.

The highest ad rate (6.1 ads/c-h) was recorded during family viewing time (Table 1). Unhealthy foods were advertised more than three times as often as healthy foods for the child viewing time and more than four times as often during family viewing time. The rates of ads for unhealthy food and beverages were significantly higher (*p* < 0.001) than those for healthy food and beverages during the child and family viewing times.

### 3.2. Food and Beverage Advertisements during Child and Family Viewing Time

During child and family viewing time (Table 2), supermarket-related ads with only unhealthy foods advertised appeared most often (0.66 ads/c-h). This was followed by fast food-related ads with unhealthy and neutral options advertised (0.55 ads/c-h). Alcohol was advertised at 0.25 ads/c-h. Of the neutral category, ads about vitamin/minerals or other dietary supplements and sugar-free chewing gum had the highest rate (0.43 ads/c-h) (Table 2).

South African Broadcasting Corporation 3 and e-TV had higher advertising rates than the other channels (7.4 ads/h and 5.3 ads/h, respectively) (Table 3). Unhealthy foods were advertised significantly more often than healthy foods especially by e-TV.

The proportion of ads with a brand benefit claim was 96%. Moreover, ads frequently used more than one brand benefit claim. As shown in Table 4, overall, brand benefit claims were used at an average rate of 3.1 claims per channel-hour (claims/ch-h) for healthy foods and 10.1 claims/ch-h for unhealthy foods. Claims promoting children or family as the users of the product, emotive claims and claims using sensory-based characteristics were the top three claims amongst healthy and unhealthy categories (Table 4). The rates of using brand benefit claims were significantly higher (*p* < 0.001) in unhealthy versus healthy products in all categories with the exception of the Suggested use (great for lunchboxes) category.

The proportion of food ads with health claims was 45%. A few of these ads made more than one health claim. The total rate of health claims recorded for the unhealthy food category (1.7 claims/ch-h) was significantly higher than the rate for the healthy food category (1.2 claims/ch-h) (Table 5). The claim of the product containing a health-related ingredient was most frequently used (0.7 claims/c-h) in unhealthy foods. The rates of using heath claims were significantly higher (*p* < 0.001) in unhealthy than healthy products for the Health-related ingredient and Nutrient comparative claim categories.

Finally, 34% of ads used power strategies. A few of these ads used more than one power strategy (Table 6). Power strategies were more significantly used in ads on unhealthy food (2.2 power strategies per channel-hour) than in ads on healthy foods (0.3 power strategies per channel-hour). Celebrity endorsement, cartoon/company owned characters and promoting the food as being child tailored (e.g., using an image of a child) were the most used power strategies. The rates of using power strategies were significantly higher in unhealthy than healthy food products where celebrated individuals and sports events were used (*p* < 0.01).

## 4. Discussion

This study investigated the exposure of South African children to unhealthy food and beverage ads. We identified 582 ads for food and beverages within the child and family viewing time. The overall rate of food and beverage-related ads was found to be 5.2 ads/ch-h. The four free-to-air TV channels advertised unhealthy foods at significantly higher rates than the healthy foods. Brand benefit claims and power strategies had significantly higher rates of use in unhealthy than healthy food ads.

### 4.1. Advertisements for Unhealthy Food and Beverages during Child and Family Viewing Times

There were almost four times as many ads for unhealthy (342: 58.8%) compared with healthy foods (90: 15.5%). This may predispose children who watch TV to choose unhealthy foods that are high in fat, salt and sugar due to their vulnerability to TV ads [5,6]. This violates the South African Marketing pledge that suggests a commitment by industry not to market food to children unless the aim is to advocate healthy dietary choices [17]. This is an indication that children in South Africa are at an increased risk of exposure to unhealthy food ads. Studies conducted in other countries have also found unhealthy foods to be proportionally more advertised than healthy foods. In Turkey, for instance, the number of ads for fast foods and beverages was found to be significantly higher than that for healthy food products [27]. In Thailand, the average ad rate of unhealthy food was also shown to be 2.9 ads/ch-h compared with the 0.2 and 0.9 ads/c-h for healthy and neutral categories, respectively [28].

Of particular concern were alcohol ads, which occurred at a rate of 0.25 ads/c-h during this period. This outcome is in violation of the SA DoH and ASA guidelines where it is clearly highlighted that no alcoholic beverages are to be shown when children are supposed to be viewing television [23]. Anderson [29] also argues that young people may be particularly susceptible to alcohol ads as they shape their attitudes, perceptions and expectancies about alcohol use. Indeed, Ausstin and Nach-Ferguson [30] found that children aged 7 to 12 years who enjoyed the alcoholic beverage ads to which they were exposed were more likely to try these beverages. Showing alcoholic beverage ads that may be appealing to children (e.g., by using celebrities and popular individuals) will more likely trigger them to become alcoholic beverage drinkers [30].

Another source of concern were the high rates of ads on sugar-sweetened beverages (SSB) because of the well documented detrimental effects they can have on children [31,32,33,34]

### 4.2. Persuasive Techniques

Persuasive techniques found in ads for all three food categories included power strategies, brand benefit claims and health claims. These techniques may be misleading as they may promote unbeneficial effects or mask harmful effects, which is of specific concern when it comes to unhealthy food [9,10]. Ads identified in the current study also carried various brand benefit claims (e.g., emotive claims, puffery) and power strategies (such as referring to famous sportspersons) that may make them more appealing. Power strategies were markedly employed to promote unhealthy foods more than healthy foods. Ads used, for example, the image of a child (child tailored) and non-sports celebrities as power strategies to promote unhealthy foods during this study. The use of cartoon characters and celebrated individuals to promote unhealthy food and alcohol are not new phenomena in South Africa. Mchiza et al. [21] had previously noted that, in 2010, 10% of the alcoholic beverage ads were shown on South African TV when children and family were supposedly watching. Mchiza et al. [21] also reported that these ads were promoted with the help of celebrated individuals such as movie actors, sportsmen and TV personalities. Delport [22] highlighted that techniques such as the use of cartoon characters are employed to create imagery of fun and excitement that appeals to children.

Oyero and Salawo [9] assert that the use of health claims when advertising unhealthy food represents a derogation of the importance of healthy foods. With the lack of intellectual capacity and skills to deal with the appeal of these messages [35], children are even more likely to fall for this deception and may easier accept these false health claims as the truth. This may shape the way they see what is healthy and unhealthy and may engrain misconceptions in their brains about what is healthy while fostering unhealthy eating habits.

Brand benefit claims were another persuasive technique utilised to advertise both healthy (3.1 claims/ch-h) and unhealthy food (10.1 claims/ch-h). Brand benefit claims have previously been used in South Africa, particularly those brand benefits that portray fun [21,36]. Mchiza et al. [21] found ads for desserts, sweets and sugar-concentrated beverages to contain portrayals of exaggerated pleasure sensations such as depictions of lovely taste, fun and addictive sensations. Pengpid and Pelzer [36] found similar claims and others such as improving one’s social worth and status. According to Harris et al. [12], the use of fun and excitement imagery in food ads has caused an increase of consumption of food in those being exposed. Repetitive exposure to these brand benefit claims tends to lead to the development of a relationship with the brand [14], which can be exploited by marketers of unhealthy foods.

### 4.3. Policy Implications

The South African Marketing to Children pledge makes it clear that there should be no use of celebrities and licensed characters (such as cartoons) in advertising unhealthy foods to children [15]. The food and beverage advertising codes (which the food companies submitted to through the pledge) asserts that children are easily influenced and so they should not be misled with false or exaggerated advertising claims [17]. Signees of the pledge are admonished to be honest in their ads and not to take advantage of the lack of experience of children or knowledge in advertising foods to them. Thus, claims such as the emotive claims recorded in this study go against the social values of advertising under the food and beverage advertising codes [17]. The common use of these strategies to advertise unhealthy foods as identified in our study violates the South African Marketing to Children pledge [17].

With the many violations of the food and beverage advertising codes and the South African Marketing to Children pledge, it appears that the outcome of the self-regulatory approach adopted by South Africa is unsatisfactory. The persistent flouting of these codes, as revealed in the current study, by Mchiza et al. [21] and by Delport [22] comes as no surprise as self-regulation around the world has proven to be ineffective in limiting unhealthy food advertising to children [26]. This unsatisfactory outcome emanates from the laxity in the enforcement of self-regulation codes [19], which could be attributed to the intention/drive among industry players to make profits. Self-regulatory policies make it appear as though advertising is being controlled while in reality all of these policies seem to do is to stifle change [19].

An introduction of statutory regulations in South Africa would signify a refreshing change in the food advertising environment. Additionally, strict monitoring and the enforcement of significant penalties may serve as a deterrent to companies and television stations who disregard the policies. The above policies have been shown to be effective in reducing unhealthy food ads to children [37]. New regulations should strictly control the use of persuasive strategies in unhealthy food ads. Educating food marketers on the importance of adhering to the policies for controlling food advertising may help bring about attitudinal change. A watershed period after which unhealthy food ads would be allowed could also be considered.

### 4.4. Strengths and Limitations of the Current Study

The strengths of the current study included the thorough and systematic assessment of ads based on a structured guide developed for international monitoring and benchmarking. The assessment also covered several domains of persuasive techniques.

The limitations included the limited scope of the current research in that the data captured were from the free-to-air TV channels only (those channels accessible to most children from disadvantaged communities) and were collected at a single point in time. As such, ads shown on other South Africa TV channels (especially those that are accessible to more affluent communities i.e., pay/subscription TV) were missed. It may therefore be impossible to generalise these data to other South African populations that have access to pay/subscription TV channels. This study was carried out on TV stations and, as such, may not adequately represent the nature of food ads in the wider food advertising space in South Africa that also includes social media ads, radio ads, etc. While the duration of the study captured periods where children are likely to be watching TV, there is the potential for children to be exposed to TV food ads outside of those hours included in this study. Therefore, the overall potential exposure for TV food ads could be higher than reported in this study. This study also did not investigate the causal effect of food advertising to South African children. For instance, in this study, only potential exposures could be assessed without accounting for the number of child viewers of these ads. As such, new research is needed that will investigate how South African children respond to food ads. The findings can be utilised for the specific regions or African countries that have access to these South African TV stations but cannot be extrapolated to countries outside this group unless they have a similar context and TV ad regulations. Lastly, our results could not be compared with earlier findings as these studies used different classification systems. We think that the classification used in the current study can serve as a benchmark for future comparisons.

## 5. Conclusions

This study suggests a high exposure among children to unhealthy food and beverage advertising including alcohol. The use of cartoons, celebrities, brand benefit claims and health claims were used more often in unhealthy versus healthy food. These techniques may foster children’s craving for unhealthy food while making unhealthy food consumption a part of their value pattern. These findings breach the South African Marketing to Children pledge and represent an unsatisfactory outcome of the self-regulation system practiced in South Africa. There is, therefore, an urgent need for a tighter control of the TV food advertising space. Options include statutory regulations and a watershed period for unhealthy food ads.

## Figures and Tables

**Table 1:** Rates of advertising (ads) during child and family viewing time.

Target Audience	Ads/c-h (Rate)					
	Total (SD)	Healthy Foods (SD)	Unhealthy Foods	Neutral	Ratio of Unhealthy Foods to Healthy Foods	*p*-Value
Child Viewing Time	4.3 (3.7)	0.8 (1.0)	2.3 (2.4)	1.2 (1.5)	3.1	<0.001
Family Viewing Time	6.1 (3.9)	0.9 (1.1)	3.8 (2.5)	1.5 (1.4)	4.4	<0.001
Average	5.2 (3.9)	0.8 (1.1)	3.1 (2.6)	1.4 (1.5)	3.8	<0.001

Legend: Mean rates of ads per channel per hour per viewing time category per food category and SD. The *p*-value resulted from testing if a difference exists between healthy and unhealthy ads in the proportion of ads with this feature.

**Table 2:** Mean food and beverage advertising rates during child and family viewing time.

	Ads/c-h (Rate)(SD)
Healthy Foods	
Bread, rice and rice products without added fat, sugar or salt, noodles	0.26 (0.53)
Oils high in mono- or polyunsaturated fats, low fat savoury sauces	0.26 (0.55)
Low sugar and high fibre breakfast cereals (<20 g sugar/100 g and >5 g dietary fibre/100 g)	0.21 (0.56)
Vegetables and vegetable products without added fat, sugar or salt	0.04 (0.21)
Milk and yoghurt (≤3 g fat/100 g), cheese (≤15 g fat/100 g) and their alternatives	0.02 (0.13)
Meat and meat alternatives	0.01 (0.09)
Supermarkets: only healthy foods advertised	0.01 (0.09)
Unhealthy Foods	
Supermarkets: only unhealthy food advertised	0.66 (0.94)
Fast food (unhealthy foods and neutral options advertised)	0.55 (0.89)
Sugar-sweetened beverages	0.34 (0.62)
Savoury snack foods (with added salt or fat)	0.28 (0.54)
Alcohol	0.25 (0.62)
Ice cream, iced confections, desserts	0.23 (0.58)
High sugar and or low fibre breakfast cereals (>20 g sugar/100 g or <5 g dietary fibre/100 g	0.15 (0.38)
Chocolate and candy	0.15 (0.38)
Sweetened bread, cakes, muffins, sweet buns	0.14 (0.38)
Fruit juice/drinks (<98% fruit)	0.11 (0.62)
Full cream milk and yoghurt	0.11 (0.41)
Meat and meat alternatives processed or preserved in salt	0.04 (0.21)
Sweet snack foods	0.03 (0.16)
High fat/salt meals	0.02 (0.13)
Neutral	
Vitamin/mineral or other dietary supplements, sugar-free chewing gum	0.43 (0.78)
Supermarkets: healthy and unhealthy foods and drinks advertised	0.35 (0.67)
Tea and coffee (excluding sweetened powder-based teas or coffees)	0.33 (0.68)
Recipe additions (including soup cubes, oils, dried herbs and seasonings)	0.17 (0.42)
Fast food restaurant (no foods or drinks advertised)	0.05 (0.23)
Baby and toddler milk formulae	0.01 (0.09)

Legend: Mean number of ads per channel per hour and SD.

**Table 3:** Mean advertisement rate by television (TV) channel.

	Ads Per Hour (Rate) (SD)					
TV Station	Total	Healthy Foods	Unhealthy Foods	Neutral	Ratio of Unhealthy Foods to Healthy Foods	*p*-Values
e-Tv	5.3 (3.7)	0.3 (0.5)	3.6 (2.9)	1.4 (1.5)	11.1	<0.001
SABC1	4.3 (3.7)	1.0 (1.3)	2.8 (2.4)	0.5 (0.6)	2.8	<0.001
SABC2	3.8 (3.9)	0.6 (0.8)	2.0 (2.2)	1.2 (1.5)	3.2	<0.001
SABC3	7.4 (7.4)	1.3 (1.2)	3.9 (2.4)	2.3 (1.6)	3.0	<0.001
Average	5.2 (3.9)	0.8 (1.1)	3.1 (2.6)	1.4 (1.5)	3.8	<0.001

Legend: Advertisement per food category per hour and SD. SABC: South African Broadcasting Corporation. E-TV: Enhanced Television. The *p*-value resulted from testing if a difference existed between healthy and unhealthy ads in the proportion of ads with this feature.

**Table 4:** Mean rates of use of brand benefit claims.

Brand Benefit Claim	Claims Per Channel-Hour (Rate) (SD)		
	Healthy	Unhealthy	*p*-Value
Sensory-based characteristic (taste, texture, appearance, aroma)	0.8 (1.1)	2.3 (2.0)	<0.001
New brand development	0.3 (0.6)	1.1 (1.4)	<0.001
Suggested use (great for lunchboxes)	0.4 (0.7)	0.5 (0.8)	0.358
Suggested users are children or the whole family	0.7 (1.1)	2.1 (1.9)	<0.001
Emotive claims (fun, feelings, popularity)	0.4 (0.8)	2.0 (1.8)	<0.001
Puffery (claiming to be advantageous over other products)	0.4 (0.7)	0.8 (1.1)	<0.001
Convenience (ready to eat)	0.1 (0.3)	0.6 (1.0)	<0.001
Price (cheap)	0.1 (0.3)	0.8 (1.2)	<0.001
Total rate	3.1 (4.6)	10.1 (9.0)	<0.001

Legend: Mean number of brand benefit claims per channel per hour by food category and SD. The *p*-value resulted from testing if a difference existed between healthy and unhealthy ads in the proportion of ads with this feature.

**Table 5:** Mean rates of health claims.

Type of Health Claim	Claims/Per Channel-Hour (Rate) (SD)		
	Healthy	Unhealthy	*p*-Value
Health-related ingredient claim	0.1 (0.4)	0.7 (1.1)	<0.001
Nutrient content claim (e.g., low fat)	0.2 (0.5)	0.4 (0.7)	0.028
Nutrient comparative claim (e.g., reduced fat)	0 (0)	0.1 (0.4)	<0.001
General health claim (e.g., healthy diet)	0.2 (0.5)	0.1 (0.6)	0.123
Nutrient and other functions (e.g., calcium, good for bones)	0.3 (0.6)	0.2 (0.5)	0.176
Reduction in disease risk claim (e.g., HF tick)	0.3 (0.6)	0.1 (0.6)	0.001
Total	1.2 (2.1)	1.7 (2.8)	0.004

Legend: Mean health claims per channel per hour by food category and SD. The *p*-value resulted from testing if a difference existed between healthy and unhealthy ads in the proportion of ads with this feature.

**Table 6:** Mean rates of power strategies.

Type of Power Strategy	Power Strategies Per Channel-Hour (Rate) (SD)		
	Healthy	Unhealthy	*p*-Value
Cartoon/company owned characters (e.g., lion)	0.1 (0.3)	0.2 (0.4)	0.185
Amateur sports person	0.0 (0.0)	0.1 (0.2)	0.031
Celebrity (non-sports) (e.g., Jamie Oliver)	0.0 (0.0)	0.3 (0.6)	<0.001
Child tailored (e.g., image of a child)	0.2 (0.6)	0.3 (0.7)	0.175
Sports events (e.g., a race)	0.0 (0.0)	0.2 (0.4)	<0.001
Total	0.3 (0.7)	1.0 (1.1)	<0.001

Legend: Mean number of power strategies per channel per hour by food category and SD. The *p*-value resulted from testing if a difference existed between healthy and unhealthy ads in the proportion of ads with this feature.

## Data Availability

Not applicable

## Abbreviations

TV	Television
SANHANES-1	The first South African National Health and Nutrition Examination Survey
NCDs	Non-communicable diseases
CGCSA	South African Marketing to Children pledge
e-TV	Enhanced Television
US	United States
WHO	World Health Organisation
ASA	Advertising Standards Authority
SABC	South African Broadcasting Corporation
SSB	Sugar-sweetened beverages
Ads	Advertisements
Ads/ch-h	Advertisements per channel per hour
Claims/ch-h	Claims per channel per hour
